# Creative Flow and Physiologic States in Dancers During Performance

**DOI:** 10.3389/fpsyg.2020.01000

**Published:** 2020-05-27

**Authors:** S. Victoria Jaque, Paula Thomson, Jessica Zaragoza, Frances Werner, Jeff Podeszwa, Kristin Jacobs

**Affiliations:** ^1^Department of Kinesiology, California State University, Northridge, Los Angeles, CA, United States; ^2^Department of Theater, York University, Toronto, ON, Canada; ^3^Department of Intercollegiate Athletics, The University of Akron, Akron, OH, United States; ^4^Cardiac Rehabilitation and Wellness, UC San Diego Health, San Diego, CA, United States

**Keywords:** autotelic experience, autonomic nervous system, dancers, flow, pre-ejection period, RMSSD

## Abstract

Pre-professional and professional dancers (*n* = 60) participated in this ambulatory psychophysiology study that investigated performance flow and heart rate and autonomic nervous system (ANS) function during three time periods: baseline rest, performance, and post-performance rest. To gather these results, the psychophysiology laboratory traveled to the concert hall to collect data on dancers. The self-report Flow State Scale (FSS) measured global flow, challenge–skill balance, sense of control, and autotelic experiences; it addresses important features of the creative experience of performing artists. These data were collected immediately following the performance. The flow measures were compared with physiologic responses to performance [heart rate, pre-ejection period (PEP), root mean square differences of successive R-R (heartbeat) intervals (RMSSD), cardiac autonomic balance, and cardiac autonomic regulation]. The regression analyses indicated that greater sympathetic nervous system (SNS) activation with performance (PEP change from base to performance) explained 8.8% of the variance in sense of control, whereas less cardiac autonomic regulation explained 13.8% of the variance in autotelic experiences. The sample was then divided into high and low flow groupings and four autonomic groups. During performance, the high autotelic group and high sense of control group had a higher distribution of dancers with co-inhibition of both ANS branches than had the low autotelic and sense of control groups who employed more co-activation of both ANS branches (chi-square analyses). These novel findings add to the growing information about the interaction of both branches of the ANS during creative performance flow states.

## IntRoduction

Capturing the physiologic creative flow experience in performers while on stage is seldom possible. Typically, creative flow assessment is conducted retrospectively. In performing artists, some of these creative flow factors include a global sense of optimal creative performance, the balance between performance challenges and skills, a sense of control (SC) while performing, and an autotelic experience (AE; valuing the performance experience for its own sake) (Hefferon and Ollis, 2006; Thomson and Jaque, 2011–2012, 2016). Performing artists give expression through unconstrained movement; their art is contingent on heightened kinesthetic sensorimotor responses. To date, ambulatory physiologic heart rate (HR) variability (HRV) assessment provides indirect observations of the autonomic nervous system (ANS) (Ellis and Thayer, 2010; Kim et al., 2018). Although this form of measurement is ambulatory, few laboratories gather *in vivo* physiologic information while performers are on stage with an audience in attendance. Seldom does the research laboratory team travel to the concert hall to study the performers. In general, research investigation is hampered by the intense demands of professional productions. Limiting factors include stage environment (backstage set up, size of wings, and type/size of stage), production elements (costumes and lighting), and professional performer/artistic team willingness to participate in research studies while performing.

This study directly assessed ANS function in pre-professional and professional dancers prior to, during, and post-performance. Creative state flow responses were gathered immediately following a public performance. Ambulatory assessment allowed a direct window into dancers’ physiology. Both the producing organization and the dancers willingly accommodated the discomfort of wearing equipment required to gather cardiorespiratory data. The goal of this study was to understand the physiologic and psychological states of flow in dancers while performing.

### Flow States in Dancers

Flow is a subjective psychological state of well-being and is strongly associated with the creative process. Reaching flow states while engaging in a preferred activity is meaningful and pleasurable (Csikszentmihalyi, 1990, 1996). Multiple factors converge during flow states, such as when the challenge of the task is matched by the skill level of the participant, when the goals are clear and feedback is unambiguous, when concentration is fully directed on the task, when the actions are executed and are merged with heightened awareness, and when the individual simultaneously has an SC without interference of self-conscious doubts (Jackson and Eklund, 2002, 2004). Often, these factors alter perceptions of time, either expanding or contracting the perceived time duration. Flow is influenced by factors that are both internally and externally directed; however, factors that are consistently present in individuals who are prone to flow experiences include possessing skills that match the challenge, effortless attention and concentration, an acute SC, and a desire to engage in activities for their intrinsic merit (Ullen et al., 2010).

Individuals who frequently experience flow states are described as possessing an autotelic personality; they desire challenge, demonstrate superior concentration skills, are intrinsically motivated, and engage in active coping strategies. They possess meta-skills such as general curiosity, persistence, and low self-centeredness (Csikszentmihalyi, 1990). These individuals generally have higher self-esteem and lower trait anxiety (Asakawa, 2010). Studies have demonstrated some associations with heritability (Mosing et al., 2012), which suggests that flow proneness is a dispositional personality trait (Ullen et al., 2012, Ullén et al., 2016). There is also an indication that more gray matter volume is found in the right caudate nucleus and that flow proneness is associated with the dopaminergic system (D2) in the dorsal striatum (de Manzano et al., 2013; Niksirat et al., 2019). These regions are associated with attention, effortless concentration, motivation, reward, impulse control, and positive affect (de Manzano et al., 2013; de Sampaio Barros et al., 2018). Personality traits associated with flow-prone individuals (autotelic personality) include low neuroticism, high conscientiousness (Ullen et al., 2012), and a hardy personality (Crust and Swann, 2013).

Studies have demonstrated that a high proportion of dancers experience heightened flow states, including possessing an autotelic personality profile (Hefferon and Ollis, 2006; Thomson and Jaque, 2011–2012, 2016). Achieving heightened flow states is associated with optimizing successful performances (Kirchner et al., 2008). These positive states are essential for dancers who must merge physical execution with creative aesthetic expression (Hefferon and Ollis, 2006). Similar to athletes, dance training promotes many of the flow dimensions, which ultimately increases optimal flow states (Swann et al., 2012). Dancers are trained to successfully engage in highly challenging performances, and this success is rooted in accomplishments such as achieving expert skill level (Edmonds et al., 2019). In order to achieve this level of performance, dancers learn to direct their attention toward the task and fully concentrate while dancing. They also learn how to set clear goals and how to appraise their achievement at sensorimotor and cognitive levels. This training enhances an SC while simultaneously diminishing self-conscious thoughts. Studies investigating flow highlight the importance of a balance between the challenge and skill level of the performer as well as the ability to effortlessly direct prolonged attention and concentration while executing a task (Swann et al., 2012; Hallet and Hoffman, 2014; Thomson and Jaque, 2016).

### Ambulatory Psychophysiology Research

The ability to non-invasively monitor cardiorespiratory function during daily tasks provides an opportunity to gather ambulatory psychophysiologic data, in particular ANS function (Wilhelm et al., 2003). The ANS facilitates physiologic adaptation. The two branches, sympathetic [sympathetic nervous system (SNS)] and parasympathetic [parasympathetic nervous system (PNS)], respectively, operate as arousing and calming physiologic regulators (Cacioppo et al., 2007). ANS patterns dynamically fluctuate in response to internal or external demands. The prototypical ANS pattern is a reciprocal interaction between the sympathetic and parasympathetic branches: as one increases activation, the other branch decreases engagement. A reciprocal pattern is most common during daily activities; however, other ANS patterns operate when energy demands increase. Co-activation or co-inhibition of both branches may occur to meet these demands. Under extreme physiologic demands, the two branches might also become uncoupled, a response that is energy efficient for short periods of time (Berntson et al., 2008). Examining the interaction patterns between the two autonomic branches (high SNS, high PNS, co-inhibition, and co-activation) can also be explained as cardiac autonomic regulation (CAR) (the total of SNS and PNS activation) and cardiac autonomic balance (CAB) (the difference between PNS and SNS activation) (Berntson et al., 2008).

Physical or psychological challenges require increased metabolic output and the complex variables contributing to HRV reflect these demands (Porges, 1995; Kim et al., 2018). Calculating HRV from cardiorespiratory measurements allows for the examination of the ANS *in vivo*. HRV is assessed by the interbeat interval, which is defined as the time period between consecutive R-to-R intervals of the heart (Porges, 1995; Kleiger et al., 2005; Kim et al., 2018). Generally, more variance between interval periods indicates greater efficiency and adaptability in the physiologic and psychological systems (Friedman, 2007; Kim et al., 2018).

### Physiological Responses and Performance Flow

To date, studies examining autonomic responses during flow states are varied; however, solid evidence supports the effectiveness of measuring HRV as a predictor of flow states (Bian et al., 2016). The pleasurable experience of flow may, in fact, be physiologically demanding and stressful. Physiologic flow states elicit more activation of the SNS, including elevated HR (Yoshie et al., 2009; Thomson and Jaque, 2011; Jaque et al., 2015), and are accompanied by increased parasympathetic withdrawal (Murch et al., 2017). Increased sympathetic tone may also operate in a “U” shape, with moderate elevation occurring during flow states but not reaching the levels found in high-arousal states such as terror and high-intensity workloads (Peifer et al., 2014; Murch et al., 2020). In contrast, evidence suggests that increased parasympathetic tone is associated with the relaxed state generated by positive flow engagement (Harmat et al., 2015); however, the investigation of vagus nerve stimulation has demonstrated that elevated parasympathetic tone diminishes creative and cognitive flexibility (Ghacibeh et al., 2006).

In several studies, it was hypothesized that both branches of the ANS may be operating (i.e., increased parasympathetic modulation of sympathetic activity) during flow states, which facilitates the task-related flow experiences and accounts for moderate HRV levels (de Manzano et al., 2010; Peifer et al., 2014; Tian et al., 2017). When reporting HRV, it is difficult to know how the two branches of the ANS are operating; however, one study identified that diminished HRV as assessed by the root mean square differences of successive R-R (heartbeat) intervals (RMSSD) was associated with heightened pleasurable flow states. This decrease in HRV was in direct response to the skill–challenge/demand compatibility necessary during flow states (Keller et al., 2011) and suggests that both branches of the ANS might be operating simultaneously (Tian et al., 2017). The complexity of the ANS is evidenced when examining both branches in relation to each other; they may interact in a complex pattern of antagonistic (reciprocal) co-activation or co-inhibition or become uncoupled (the most energy-efficient strategy for high-demand situations) (Berntson et al., 2008).

When examining the physiologic effects of dance, it is important to consider level of expertise and performance conditions. For example, dance expertise is generally associated with greater physiologic efficiency cultivated by increased training load. Dancers, like athletes, have chronic training adaptations in HRV owing to increased capacity of the parasympathetic branch, which reflects in increased HRV (da Silva et al., 2015). As well, exercise training-adaptations include an enhanced parasympathetic drive to the sinoatrial node of the heart and a faster rate of recovery (specifically a faster restoration of the parasympathetic response as measured by RMSSD) (Danieli et al., 2014; Sala et al., 2015). These physiological adaptations allow elite performers, who achieve flow states during a public performance, to increase sympathetic activation, as evidenced by increased HR and perspiration rate, and to decrease parasympathetic activity while performing (Nakahara et al., 2009). Enhanced sympathetic activation and reduced parasympathetic activation are also evident prior to a competition or performance season; this evidence further demonstrates the unique ANS responses necessary to meet heightened physiologic demand (Edmonds et al., 2019). These changing responses in the ANS are related to greater HRV over a protracted period of time (de Manzano, 2010; de Manzano et al., 2010). It is also important to note that both the subjective experience of flow and the subsequent physiologic responses are highly influenced by individual aesthetic responses; one dancer may be highly aroused by a particular musical and choreographic work, whereas another dancer may be bored by the same piece of music and choreography (Salimpoor et al., 2009). Ultimately, onstage subjective flow experiences offer opportunities to match elite skills with high performance challenges, including a heightened SC and deep concentration (Robb and Davies, 2015); and these states require autonomic adaptations.

### Measuring Autonomic Responses

Autonomic function can be assessed *via* time-domain or frequency-domain variables. In this study, two time-domain variables, pre-ejection period (PEP) and root mean square differences of successive R-R (heartbeat) intervals (RMSSD), were examined. This decision was based on recommendations by the Task Force of Cardiology as well as previous studies indicating that these variables provide the most accurate measure of the sympathetic and parasympathetic branches, respectively (Task Force of the European Society of Cardiology, and the North American Society of Pacing, and Electrophysiology, 1996; Cacioppo et al., 2007; Wang and Huang, 2012; Sztajzel, 2014). PEP is associated with SNS activity. It is the period between left ventricle stimulation and the opening of the aortic valve (Berntson et al., 2008). A longer time period prior to aortic valve opening is associated with decreased activation of the SNS. When measuring PEP, values are inversely related to sympathetic activation (Berntson et al., 2008; Murch et al., 2020). RMSSD is the root mean square differences of successive R-R (heartbeat) intervals. It is generally regarded as a stable measurement that reflects alterations in autonomic tone that is predominately vagus mediated (parasympathetic) (Hill et al., 2009; Wang and Huang, 2012; Sztajzel, 2014; Shaffer and Ginsberg, 2017). Like most PNS measures, RMSSD is influenced by respiratory rate as well as potential SNS influences, which may limit conclusive findings when it is used as an index of vagal cardiac control (Berntson et al., 2005).

Respiration influences ANS activity: inspiration diminishes vagal tone input to the heart and increases HR, whereas exhalation increases vagal tone and decreases HR. These fluctuating patterns reflect optimal HRV (Cacioppo et al., 2007). Several other factors influence ANS activity, and in particular HRV. These include physical conditioning, especially conditioning prior to major sport or dance performances (Edmonds et al., 2019). Measuring participants when they are exercising directly influences HRV measurement, even if HR remains consistent; thus, all ambulatory assessment is limited by the influence of even light exercise (Weippert et al., 2015). HRV is diminished as individuals age (Zhang, 2007; Tegegne et al., 2018), and female sex is associated with diminished HRV (Tegegne et al., 2018).

Examining flow states via the respiratory and cardiac systems during activities that are mentally engaging demonstrates clear patterns of increased HR and respiratory volume; however, studies indicate that they are generally moderately increased, which demonstrates effortless concentration and attention (Ullen et al., 2010; de Sampaio Barros et al., 2018). During physical activity such as dance, dancers’ HR and respiratory rate/volume are much greater during performance compared with their resting states. These ANS responses are much greater compared with ANS values in participants who are engaging in mental challenges such as computer games (Tian et al., 2017; Murch et al., 2020). This dynamic range in dancers will influence HRV measurements, which makes comparisons across different studies challenging.

### Study Purpose

The current institutional review board (IRB)-approved study investigated dancers’ physiologic responses in a performance setting, as well as flow variables [global flow, challenge–skill balance (CSB), SC, and AE] that are associated with creative performance experiences. We first hypothesized that dancers would generally experience high flow states while performing (Hefferon and Ollis, 2006; Thomson and Jaque, 2016). Further, dancers who experienced high global flow and two performance-related flow dimensions (CSB and SC) would mobilize more sympathetic activation because performance demands would require increased cardiorespiratory activation. We also hypothesized that increased autotelic state flow would be associated with more ANS modulation. The goal of this study was to examine activation patterns in both branches of the ANS during a high-flow performance activity.

## Materials and Methods

### Participants

A subsample of 60 dancers was selected from an ongoing larger psychophysiology study. Inclusionary criteria included the following: (1) all dancers who have trained 5 or more years, (2) they worked professionally (remunerated for their performance) in at least one production, (3) they were scheduled to perform in a public concert hall, and (4) they were 18 years of age or older. There were 40 (66.7%) female and 20 (33.3%) male dancers, ranging in age between 18 and 37 years, with a mean age of 22.20 (*SD* = 3.99). This sample had a diversity distribution of the following: 12 (20.7%) Black, 16 (27.6%) Asian, 19 (32.8%) Caucasian, and 11 (19.0%) Latinx (2 were missing ethnicity data).

### Study Procedure

The dancers were invited to participate in the study. Voluntary written informed consent was provided after a brief introduction to study goals and requirements. Once entering the study, the dancers completed a large research package, containing multiple psychological and health history self-report measures. They were then fitted into the VivoMetrics LifeShirt (Ventura, CA, United States), an ambulatory measuring instrument with high HRV accuracy (Heilman and Porges, 2007). Once fitted and connected to the VivoMetrics LifeShirt, dancers participated in one rehearsal session. This rehearsal session provided an opportunity for the dancers to gain comfort wearing the ambulatory physiologic measuring equipment. It was posited that increased familiarity would decrease distraction during a public performance, hence approximating performance responses. All dancers in this study spent several months in preparation for the dance concert; they were physically and psychologically prepared for a series of public performances and standard performance preparations were maintained throughout the data collection process. Maintaining rehearsal and performance conditions ensured that dancers would be physiologically evaluated with minimal interference resulting from the ambulatory physiologic data collection process.

In both the rehearsal and performance situations, the same research preparatory and follow-up procedures transpired. First, three electrodes were attached to the dancers (two electrodes on the upper right and left chest wall and one on the left abdomen). Preparation for electrode placement included cleaning the skin with alcohol swabs, mildly abrading the skin to secure electrodes, and, for some male participants, shaving chest hair. Once the VivoMetrics LifeShirt system was securely fastened, respiratory calibration was conducted. Calibration involved seven forced exhale/inhale breath cycles into a fixed calibration bag; this sequence was repeated twice in a protocol that included both the seated and standing positions. During calibration, a nose clip was placed on the dancers to stop breathing through the nose. Once the VivoMetrics LifeShirt was calibrated, the dancers rested in a supine position (legs and arms uncrossed) for 7 min to collect baseline resting physiologic data. The dancers were then free to prepare for their upcoming performance as usual, including performing in a medium-sized concert hall. When the performance ended, the dancers answered a state flow self-report scale and then completed a 7-min post-performance rest session, once again in a supine position. This post-rest epoch provided physiologic data related to recovery capacity post-performance. Completion of the post-performance rest was followed by removal of the LifeShirt and any debriefing requested by the dancers regarding the research study.

While wearing the LifeShirt, the dancers were continually followed by a research assistant who completed a written event diary. The intention of the diary was to provide a timeline record of all observed behaviors of the dancers (i.e., walking, sneezing, practicing, laughing, chatting with others, bathroom visits, eating, and dressing). These behaviors all influence ANS reactivity and add invaluable information regarding performer pre-performance practices. The research assistant also followed the dancers into the wings and gathered observational information regarding performance activity while the dancers were on stage. Video recording of the performance provided further information about the performance.

### Measurements

#### VivoMetrics^TM^ LifeShirt

The ambulatory VivoMetrics LifeShirt system is a non-invasive multi-sensory monitoring system that collects and stores cardiopulmonary data (Wilhelm et al., 2003). The entire system includes a vest, electrodes, an accelerometer, a data cable, and a portable recorder. Within the vest, two respiratory sensor bands wrap around the ribcage and abdomen of the dancers. These two sensor bands provide respiratory inductive plethysmography information reflecting respiratory function such as tidal volume and respiratory rate. Between the respiratory bands is a thoracocardiography (TCG) band that gathers left ventricular volumetric changes. Further cardiac data are gathered from the embedded three-lead EKG data system. Accurate sizing of the vest is essential in order to ensure TCG band placement around the chest at the xiphoid process. Embedded in the LifeShirt is a tri-axial accelerometer that provides information related to posture and activity levels. A data cable was connected to the vest (attached via Velcro) and was connected to the LifeShirt portable recorder (compact memory card captured recorded data). The 2-lb recorder was placed into a small fanny pack that was secured around the dancers’ waist. All leads were secured with adhesive tape to reduce artifact due to lead wire movement.

Once data collection was completed, the data cable was removed and the data were imported into a desktop computer equipped with the VivoLogic^®^ v3.2 software system. This system filters respiratory and cardiac signal noise, although manual EKG cleaning is also required (Wilhelm et al., 2003). Each heartbeat is evaluated to determine if it is a normal beat or an ectopic beat or whether it includes a P wave abnormality, T wave abnormality, QRS abnormality, and/or pre-ventricular contraction (PVC). All heartbeat abnormalities were annotated and either removed from further statistical analyses or interpolated to maintain continuity in the data (Morelli et al., 2019). The Vivologic software computes temporal variables such as respiratory rate, HR, tidal volume, respiratory sinus arrhythmia (RSA), PEP, standard deviation of R-R intervals (SDNN), root mean square differences of successive R-R (heartbeat) intervals (RMSSD), ands spectral frequency analyses to derive variables such as normalized high frequency (HFn), normalized low frequency (LFn), and very low frequency (VLF) HRV and the ratio of LF:HF. In this study, only HR, PEP, RMSSD, CAB, and CAR are reported.

#### Flow State Scale-2

The Flow State Scale-2 (FSS2) (Jackson and Eklund, 2004) is a 36-item, self-report instrument that assesses the construct of state flow. A five-point Likert scale ranging from 1 (*no agreement)* to 5 (*strongly agree*) is used. The FSS2 captures subjective flow experiences immediately following the completion of an activity. Mean scores are calculated for the nine dimensions of flow (each with four items), plus a mean global flow score is calculated. In this study, global flow (α = 0.956) and three subscales were included: a) CSB (α = 0.917), b) SC (α = 0.927), and c) AE (α = 0.909). The flow scale scores can be divided into the following: first, low agreement (mean scores ranging between 1 and 2), which suggests that the person’s experience was not flow-like in nature; second, moderate level (mean scores ranging between 2 and 4), indicating some endorsement of flow experiences; and lastly, high level (mean scores ranging between 4 and 5), indicating the respondent strongly agreed with the flow experience in their selected activity. There is adequate reliability, construct validity, and internal consistency in the FSS2 (Jackson and Eklund, 2002, 2004; Jackson et al., 2008). It is considered to be an excellent measure of state flow experiences.

### Study Analyses

All statistical analyses were conducting using SPSS 26. First, descriptive statistical analyses were conducted (see Table 1 for mean and standard deviations). The dancers were then divided into two groups on the basis of a median split for scores on the four FSS2 scales: (1) global scale, (2) CSB, (3) SC, and (4) AEs. Prior to further statistical analyses, the data were normalized to their square root values (flow variables) and the natural logs for all physiologic variables (HR, PEP, and RMSSD). The three physiologic variables (HR, PEP, and RMSSD) were examined in three epochs: pre-performance base rest, performance (5–9 min during the first dance performed in the concert), and post-performance rest period. Changes were then calculated between each of the three epochs (base rest to performance, performance to post-rest, and base rest to post-rest). According to Berntson et al. (2008), four categorical ANS groups can be created based on *z*-scores for sympathetic and parasympathetic measures. For this calculation, we multiplied PEP*z* by −1 for SNS and used RMSSD*z* for PNS. On the basis of PEP and RMSSD performance values, the dancers were divided into four categorical ANS groups to represent (1) co-activation of the SNS and PNS (-PEP*z* and RMSSD*z* > 0), (2) high SNS activation (−PEP*z* > 0 and RMSSD*z* ≤ 0), (3) co-inhibition of the SNS and PNS (-PEP*z* and RMSSD*z* ≤ 0), and (4) high PNS activation (RMSSD*z* > 0 and −PEP*z* ≤ 0). A crosstab chi-square analysis was calculated to compare the four ANS groupings with the four flow groups (global, CSB, SC, and autotelic). Lastly, a series of exploratory stepwise linear regression analyses were conducted to determine physiologic predictors (PEP change from base to performance, RMSSD change from base to performance, CAB performance, and CAR performance) for state global flow, CSB, SC, and autotelic flow in the full sample of dancers. A series of linear regression analyses were also conducted to determine only how the flow dimensions (CSB and SC) predicted the variance in global flow and the autotelic flow dimension.

**TABLE 1:** Mean descriptive statistics and standard deviations (SDs).

	HR (bpm)	PEP (ms)	RMSSD
Base rest	71.63 (9.64)	129.17 (18.65)	52.48 (31.83)
Perform	157.48 (18.12)	87.58 (20.81)	12.50 (7.76)
Post-rest	87.99 (11.96)	128.63 (19.46)	28.24 (22.70)
Δ Base - Perform	86.35 (19.69)	−40.60(24.08)	−40.34(31.39)
Δ Base - Post	16.45 (11.71)	0.45 (19.39)	−24.60(30.62)
Δ Perform - Post	69.89 (18.74)	−41.05(20.01)	−15.74(22.83)

*bpm, beats per minute; HR, heart rate; PEP, pre-ejection period; RMSSD, root mean square differences of successive R-R (heartbeat) intervals.*

## Results

Descriptive statistics are reported in Table 1 for physiologic raw mean values and standard deviations. Descriptive statistical results for the four flow scales are as follows: (1) global flow (*M* = 4.15, *SD* = *0.54*, minimum = 2.75 and maximum = 4.86, *mdn* = 4.19), (2) CSB (*M* = 4.36, *SD* = *0.71*, minimum = 1.50 and maximum = 5.00, *mdn* = 4.50), (3) SC (*M* = 4.30, *SD* = 0.74, minimum = 2.25 and maximum = 5, *mdn* = 4.50), and (4) AE (*M* = 4.49, *SD* = 0.74, minimum = 2.00 and maximum = 5.00, *mdn* = 4.75). The global, CSB, SC, and AE groups were then created based on a median split derived from the participant data for these four flow scales.

CAB (difference between PNS and SNS) and CAR (total of SNS and PNS activation) were calculated (Berntson et al., 2008) for performance (CAR: *M* = 0.04, *SD* = 1.34 and CAB: *M* = −0.01, *SD* = 1.48). These variables were examined as separate measures to evaluate response patterns in the two branches of the ANS. They were included in the regression analyses.

Because RMSSD decreases with advancing age (Zhang, 2007; Tegegne et al., 2018), a correlation analysis was conducted to determine a relationship between age and all physiologic variables. There were no significant relationships between age and CAB, CAR, PEP, and RMSSD at base rest, performance, and post-rest; however, when examining percentage changes between base rest and performance, only age and RMSSD were low but significantly related (*r* = *0.293*, *p* = 0.022).

A crosstab chi-square analysis investigated the four ANS patterns during the public dance concert performance. There were no group differences for global flow (*p* = 0.245) and CSB (*p* = 0.246); they were equally distributed amongst the four ANS categories. There were differences in the high/low SC groups and four ANS categories [χ^2^(3, 1) = 8.402, *p* = 0.038]. The low SC group engaged more co-activation and SNS dominant activation and less co-inhibition during performance, whereas the high SC group had the inverse relationship, with more co-inhibition during performance and less co-activation and SNS dominance. There were also differences in the high/low AE groups and four ANS categories [χ^2^(3, 1) = 8.811, *p* = 0.032]. The low AE group engaged more co-activation and less co-inhibition during performance, whereas the high AE group had the inverse relationship, with more co-inhibition during performance and less co-activation of both SNS and PNS.

A series of four stepwise linear regression analyses were conducted to explain the physiologic variances in (1) global flow, (2) CSB, (3) SC, and (4) AE. In these analyses, the predictor variables included age, CAB, and CAR during performance and the changes from base to performance PEP_Ln and RMSSD_Ln.

1. When global flow was the dependent variable, no variables were significant predictors of global flow.
2. Likewise, when CSB was the dependent variable, no variables were significant predictors of CSB.
3. When SC was the dependent variable, the change in PEP_Ln from base to performance explained 8.8% of the variance [*F*(1, 59) = 5.683, *p* = 0.020, β = 0.296, with *R*^2^ of 0.088]. Age, performance CAB, CAR, and RMSSD_Ln change from base to performance were not significant predictors of the variance in SC.
4. When AE was the dependent variable, CAR performance explained 13.8% of the variance, although in a negative direction [*F*(1, 59) = 9.421, *p* = *0.003*, β = −0.371, with *R*^2^ of 0.138]. Age, performance CAB, and the changes in PEP_Ln and RMSSD_Ln from base to performance were not significant predictors of the variance in AE. This finding demonstrates less co-activation of both branches of the ANS during autotelic flow, supporting the chi-square results.

Lastly, two regression analyses were conducted to determine how the flow dimensions predicted global flow (CSB, SC, and AE) and how CSB and SC predicted the variance in the AE during performance. (1) When global flow was the dependent variable, SC explained 73.4% of the variance [*F*(1, 58) = 160.002, *p* < 0.001, β = 0.520, with *R*^2^ of 0.734], whereas CSB added another 11.4% to the explanation of the variance [*F*(1, 57) = 42.927, *p* < 0.001, β = 0.477, with Δ*R*^2^ of 0.114]. Together, SC and CSB explained 84.8% of the variance in global flow. AE (*p* = 0.634) was not a significant predictor of the variance in global flow during performance. (2) When AE was the dependent variable, CSB explained 56.6% of the variance [*F*(1, 58) = 75.607, *p* < 0.001, β = 0.752, with *R*^2^ of 0.566]. SC (*p* = 0.057) was not a significant predictor of the variance in AE.

## Discussion

Professional and pre-professional dancers are highly skilled performers who have dedicated time and effort to realize their level of expertise. One of the study goals was to investigate flow states in dancers; in particular, the flow variables associated with creative performance experiences. The findings in this study demonstrated that dancers experienced moderate to high flow states, with many claiming that they strongly agreed with the flow-like statements when dancing in the concert hall. It is important to recognize that the mean score for the full sample of dancers was the highest for AEs (*M* = 4.49), with CSB (*M* = 4.36) and SC (*M* = 4.30) slightly less and global flow the least (*M* = 4.15). Global flow was included in this study to capture the complexity of flow experiences. It is derived from all nine dimensions of flow; however, these dimensions contribute unevenly to this score (Jackson and Eklund, 2004). Dance, as a performing art, demands a complex integration of factors such as music, lights, costumes, other dancers, and sensorimotor coordination. Heightened attention during a successful performance is associated with the complexity of global flow (de Sampaio Barros et al., 2018). The decision to examine CSB and SC was based on the fact that the dancers in this study had substantial expertise and training and that they were fully prepared to perform after months of rehearsal preparation (Edmonds et al., 2019). We believed that these training factors would contribute strongly to the flow dimensions of CSB and SC. AE, the scale that measures flow proneness, was equally important in this study, as evidenced by the fact that the dancers in this study all volunteered to perform in the concert without remuneration. It was not surprising that this group endorsed elevated AEs; most dancers are intrinsically motivated to participate in this art form and derive profound pleasure from this artistic medium (Hefferon and Ollis, 2006). In this dancer sample, an SC and CSB were strong predictor variables for global flow (84.8%). CSB strongly predicted AEs (56.6%). These results suggest that CSB and SC greatly contribute to dancers’ flow proneness (Hefferon and Ollis, 2006).

Investigating physiologic variables while dancers performed in a concert hall with an audience present provided *in vivo* data. The general physiologic pattern for all dancers demonstrated that HR increased while dancing and never fully returned to base-rest levels during the post-rest session. This pattern is not surprising. Any physical activity will increase HR, and the level of HR reached by the dancers was considered to be in the range of moderate–high workload levels (Inesta et al., 2008). As well, more SNS activation was evident while dancing (decreased PEP). This was validated in the regression analyses that indicated more SNS activation (PEP change from base to performance) in dancers who experienced greater SC while performing. This finding was similar to a study that investigated electronic gaming use and PEP influences during flow states (Murch et al., 2020). In order to meet physical exertion demands, vagal tone must diminish with SNS activation, a reciprocal pattern that was evident in the dancers. This physiologic finding supports a study that demonstrated elevated SNS responses in non-dancers who executed spontaneous movements with and without music (Bernardi et al., 2017). However, in the Bernardi et al. (2017) study, the effect of music increased PNS modulation of HR, a contradictory finding to the dancers in this study who performed onstage (with music), as well as the study that examined dancers baseline PNS responses during a week of performances (Edmonds et al., 2019). Dancers also had reduced SNS activation during post-rest, a pattern that resembles performance to recovery responses in elite athletes (da Silva et al., 2015). However, HR and RMSSD did not return to base-rest levels during the post-rest session, which may explain the elevated HR despite a return of PEP to pre-performance rest levels. Another explanation for this pattern might be associated with the reality that performers are eager to meet family, friends, or fans at the conclusion of a performance. They generally remain emotionally excited, and it is during this emotional state that they also completed the state flow scale, which might explain their high flow scores and the different physiologic levels during post-performance rest.

A major question in this study and other research studies is how the two branches of the ANS interact during flow states. In the chi-square analyses, there were no distinct patterns between the two branches for the high and low global flow and CSB groups. Dancers were equally distributed between the co-activation, high SNS, co-inhibition, and high PNS during performance. This finding suggests that CSB and global flow may provoke different physiologic strategies, depending on the dancers. Studies that indicate that global flow and CSB are associated with less SNS activation may not be accounting for the complex interactions of the ANS branches (Tozman et al., 2015).

A different pattern emerged for the SC groups. Although the regression analysis indicated that the percent change in SNS from base rest to performance was a significant predictor of SC flow (8.8%), the chi-square analyses offered a more complex picture. In the low SC group, more co-activation of both ANS branches or more SNS predominant activation was evident. They also had less co-inhibition of both ANS branches during performance. The high SC group had the inverse relationship, with more co-inhibition of both ANS branches during performance and less co-activation of both branches and SNS dominance. This result indicates that dancers who experience a heightened SC were able to inhibit both branches of the ANS, a finding that supports a study that demonstrated that increased PNS arousal was associated with a more relaxed state during flow experiences (Harmat et al., 2015).

When examining autotelic flow proneness, the regression analysis indicated that reduced CAR (total of SNS and PNS responses) was a predictor for AEs. The chi-square analyses supported this finding for the high AE group. The low AE group engaged in more co-activation and less co-inhibition of both branches of the ANS during performance, whereas the high AE group had the inverse relationship, with more co-inhibition during performance and less co-activation of both SNS and PNS. These results suggest that dancers who are flow-prone (AE) inhibit both the SNS and PNS branches during performance, which may influence effortless concentration and heighten kinesthetic awareness, a finding that resembles the Bian et al. (2016) investigation on physiologic indicators of flow states. It may also promote an embodied SC during preparation for flow experiences (Edmonds et al., 2019).

The limitations of this study include the challenges of filtering EKG noise while the dancers performed. Movement artifact interferes with clean EKG signals; however, signals were carefully cleaned and interpolated in this study; consequently, data were preserved as a result of this EKG cleaning process. More sophisticated physiologic recording instruments are currently available, which may decrease signaling nose. Future studies conducted on newer ambulatory systems are recommended to confirm the findings in this study. A second limitation is the high variability in physiologic measures; RMSSD is influenced by respiratory rate, which limits conclusive findings related to vagal cardiac control (Berntson et al., 2005), although it is important to note that RMSSD is less influenced by respiratory rate compared with HRV-RSA measurements (Hill et al., 2009). All forms of movement and posture changes directly influence HRV responses, as well as respiration patterns (Sipinkova et al., 1997; Weippert et al., 2015). These factors may influence the physiologic responses more than the actual flow experience while dancing, a reality that may significantly inhibit the reporting of physiologic responses associated with flow states. A third limitation is sample size, although most psychophysiology studies examine sample sizes smaller or similar to this study (Edmonds et al., 2019). A larger sample would allow more in-depth analyses with other psychophysiological variables. Many physiologic variables (respiratory and HRV derived from spectral frequency analyses) were excluded from analyses in the current study in an effort to maintain sufficient statistical power. Fourth, age and gender (more so in women) negatively influence HRV, and these factors may have confounded the research findings (Zhang, 2007; Tegegne et al., 2018). The majority of the dancers were females and in their twenties, which may have diminished RMSSD levels, although the correlation analyses demonstrated no significant association between age and any of the physiologic variables, with the exception of percent change between base rest and performance for RMSSD (*r* = 0.293, *p* = 0.022). Fifth, the initial research design failed to measure dancers in an upright position, which compromises the examination of postural influences on HRV. Postural changes may provoke physiologic changes, which diminishes confidence in determining physiologic changes due to flow characteristics (Sipinkova et al., 1997; Weippert et al., 2015). This is a clear limitation in the study design, and future studies should measure base-rest in supine and seated positions as well an ambulatory epoch in which the dancers are just walking backstage. This would isolate the physiologic influence of movement in general compared with during a dance performance. Lastly, self-report flow measures contain subjective biases. Future recommendations include comparing rehearsal responses to performance, preparation in the wings, and gathering data of dancers standing and moving backstage as they prepare for a public performance. This information may demonstrate psychological and physiologic differences between these conditions (rehearsal/performance and standing/dancing). In this study, the rehearsal data were excluded based on the fact that the dancers required time to habituate to the ambulatory equipment. This habituation process may confound the assessment of rehearsal physiologic responses, hence limiting the reliability of the rehearsal data that were collected. Lastly, it is suggested that examining performers in other performance domains while they perform in concert settings may provide different physiologic responses during flow states.

Flow is a positive experience that is highly correlated with creative engagement (Csikszentmihalyi, 1990, 1996, 1999; Robb and Davies, 2015). Talented individuals, such as performing artists, are recognized as novelty seeking, persistent, highly focused, and possessing abilities for aesthetic and emotional expressions (Nakahara et al., 2010). These traits are also inherent in highly creative individuals who frequently enter flow-like states while engaging in a creative process (Teng, 2011; Mosing et al., 2012; Ullen et al., 2012). In this study, dancers endorsed high autotelic flow, CSB, SC, and global flow; SC and CSB dimensions were strong predictors (84.8% of the variance) of global flow during the performance. Perhaps some of their success as dancers resides in a dynamic physiologic ANS. A unique factor in this study was the examination of the four ANS patterns (co-activation of both ANS branches, co-inhibition of both ANS branches, SNS dominant, and PNS dominant) and their relationship to flow dimensions. Dancers who experienced high autotelic flow and high SC were able to co-inhibit both SNS and PNS branches while performing. Consequently, dancers may derive confidence and pleasure that are rooted in their ability to effectively meet physiologic performance demands (Peifer et al., 2014).

## Data Availability Statement

The datasets generated for this study are available on request to the corresponding author.

## Ethics Statement

The studies involving human participants were reviewed and approved by the California State University, Northridge Human Subjects Committee. The patients/participants provided their written informed consent to participate in this study.

## Author Contributions

SJ and PT were co-principal investigators and participated in the data collection, data processing, SPSS data entry, data analyses, and manuscript writing. JZ, JP, FW, and KJ participated in the data collection, data processing, and SPSS data entry.

## Conflict of Interest

The Performance Psychophysiology Laboratory at California State University, Northridge, Department of Kinesiology, receives no funding beyond departmental support for laboratory access and computer software access. The authors declare that the research was conducted in the absence of any commercial or financial relationships that could be construed as a potential conflict of interest.
